# Resveratrol inhibits the formation of *Staphylococcus aureus* biofilms by reducing PIA, eDNA release, and ROS production

**DOI:** 10.3389/fvets.2025.1594239

**Published:** 2025-04-30

**Authors:** Jinfei He, Yilong Cui, Yan Liu, Jingdong Mao, Yanxin Dong, Ruizhi Yao, Dahan Yang, Peichao Fan, Jiangdong Xue

**Affiliations:** ^1^College of Animal Science and Technology, Inner Mongolia MINZU University, Tongliao, China; ^2^College of Life Sciences and Food Engineering, Inner Mongolia MINZU University, Tongliao, China

**Keywords:** resveratrol, *Staphylococcus aureus*, biofilm, polysaccharide intercellular adhesion, extracellular DNA, reactive oxygen species

## Abstract

**Introduction:**

*Staphylococcus aureus* is a zoonotic pathogen that is difficult to control. Resveratrol (RES) has been shown to have significant antibacterial effects. The present study aimed to investigate the inhibitory effect of RES on the formation of *Staphylococcus aureus* biofilms and their molecular mechanism.

**Methods:**

First, the minimum inhibitory concentration and inhibitory action curve of RES against *Staphylococcus aureus* were obtained through testing. Second, we found that RES can inhibit biofilm formation by reducing the release of polysaccharide intercellular adhesion (PIA) and extracellular DNA (eDNA) from *Staphylococcus aureus*.

**Results:**

RES treatment significantly reduced the production of reactive oxygen species (ROS) and nicotinamide adenine dinucleotide phosphate (NADPH) in *Staphylococcus aureus*, indicating that ROS and NADPH are closely related to biofilm formation.

**Conclusion:**

This study demonstrates that RES inhibits the formation of *Staphylococcus aureus* biofilms by reducing PIA, eDNA release, and ROS production, and these results provide new ideas for the clinical application of RES in the treatment of *Staphylococcus aureus* infection.

## 1 Introduction

*Staphylococcus aureus* is an important zoonotic pathogen and has become one of the most common pathogens of bacterial food poisoning and hospital- and community-acquired infections (1). It can cause infections ranging from mild skin and soft tissue infections to life-threatening endocarditis, pneumonia, and bacteremia, posing a serious threat to body health (2). Due to the extensive and unreasonable use of antibiotics, the emergence of drug-resistant strains of *Staphylococcus aureus* has posed greater challenges to clinical treatment (3). Between 1990 and 2021, methicillin-resistant *Staphylococcus aureus* (MRSA) caused the most significant increase in antimicrobial resistance-related deaths worldwide, with an increase in the total number of deaths from 83,300 to 680,000 (4). Prevention and control *of Staphylococcus aureus* has become an important focus of attention.

*Staphylococcus aureus* generally has two states: floating state and coated state (5). Antibiotics will kill some sensitive floating-state and metabolically active bacteria, but due to the encapsulation of biofilm, *Staphylococcus aureus* can escape the natural defenses of the host and quickly acquire antibiotic resistance (6). Biofilm is a polymer secreted by bacteria outside the cell. The main components are similar to the intracellular components of microorganisms, wrapped in the surface of the bacteria, and can protect the bacteria from the external environment, the host immune system, and other adverse conditions (7). Therefore, the bacteria in the biofilm can usually show a strong tolerance to environmental pressure, and the tolerance of the same bacteria to antibiotics in the biofilm state is several times greater than that in the floating state (8).

*Staphylococcus aureus* attached to the tissue surface synthesized various extracellular polymers at the same time as proliferation. With the help of extracellular polymers, bacteria adhered to each other and aggregated and subsequently formed gradually mature and stable biofilms (9). Polysaccharide intercellular adhesion (PIA) is a special polysaccharide antigen mediated by which bacteria adhere to each other, then differentiate and proliferate, form a multilayer cell mass, and finally produce a large amount of mucus to promote biofilm formation (10). The biosynthesis of PIA is encoded in the ica locus and contains four functional genes, *icaA*, *icaB*, *icaC*, and *icaD*, which are necessary operons for biofilm formation (11). Extracellular DNA (eDNA) is a nucleic acid component that exists outside the cells of bacteria in biofilms (12). *Staphylococcus aureus* can release eDNA in various ways to support biofilm formation. In addition, *cidA* and *cidB* are important factors that control bacterial lysis and eDNA release and thus participate in biofilm formation (13). *SarA* is another important regulatory gene affecting cell wall adhesion. The SarA protein family expressed by the coregulator promotes adhesion and early biofilm formation by inhibiting nucleic acid cleavage and extracellular enzyme activity (14). Therefore, the formation of bacterial biofilm is regulated by many factors, and reducing the expression of these factors can effectively inhibit biofilm formation.

Reactive oxygen species (ROS) are a class of highly reactive substances formed after the electrons of ground-state oxygen molecules are acquired. In bacterial biofilms, ROS induces genetic variation, promotes cell death in specific biofilm regions, and regulates biofilm development (15). Studies have shown that ROS is an indispensable factor involved in the regulation of bacterial biofilm formation and virulence gene expression, and the ROS production of *Staphylococcus aureus* is significantly increased during biofilm formation (16, 17). Nicotinamide adenine dinucleotide phosphate (NADPH) is involved in regulating these processes by catalyzing the production of ROS (18). Therefore, reducing ROS production is an effective approach to explore the inhibition of *Staphylococcus aureus* biofilm formation.

Resveratrol (RES) is a natural polyphenol antioxidant that can reduce deltamethrin-induced oxidative damage by upregulating the expression of Nrf2 (19) and has obvious antioxidant effects. In addition, RES, as a key bioactive ingredient of resina draconis, can play an important role through its significant antibacterial activity (20). Res treatment enhances NF-κB-p65 deacetylation and reduces inflammatory activity in a Sirt1-dependent manner, providing novel insights into Cr (VI) detoxification (21). However, there are few reports on the use of resveratrol in the field of anti-*Staphylococcus aureus* biofilm formation (22). In this study, the role and mechanism of RES in inhibiting the formation of *Staphylococcus aureus* biofilms were discussed, providing a new reference and direction for the clinical treatment of *Staphylococcus aureus* infection.

## 2 Materials and methods

### 2.1 Bacterial strains and drugs

*Staphylococcus aureus* standard strain 25923 was purchased from the American Type Culture Collection (ATCC, Rockville, MD). RES, doxycycline, oxacillin, and ceftriaxone were purchased from Shanghai Yuanye Biotechnology Co., Ltd. (Shanghai, China).

### 2.2 Culture of *Staphylococcus aureus*

*Staphylococcus aureus* was inoculated in tryptic soy broth (TSB) (Thermo Fisher Scientific, United States) and cultured at 300 r/min at 37°C for 18 h. The suspensions were then adjusted with TSB to 1 × 10^6^ CFU/mL for subsequent detection.

### 2.3 Detection of minimum inhibitory concentrations

The minimum inhibitory concentration (MIC) of RES and the three drugs against *Staphylococcus aureus* were tested according to the procedure described in the Clinical and Laboratory Standards Institute (CLSI) guidelines.

### 2.4 Detection of the bacteriostatic curve

Based on the MIC results, the bacterial solution concentration was adjusted to 1 × 10^6^ CFU/mL, and 100 μL of bacterial solution was added to the 96-well culture plate. Then, various drugs were added at concentrations of 2 MIC, 1 MIC, 1/2 MIC, and 1/4 MIC and cultured at 37°C for 0, 4, 8, 12, 16, 20, and 24 h. The OD600 value was detected using an enzyme labeler (Thermo Fisher Scientific, United States), and the curve was drawn. There were six repetition holes per concentration, and the experiment was independently repeated three times.

### 2.5 Detection of biofilm formation

*Staphylococcus aureus* was cultured in 96-well culture plates for 6, 12, 24, and 36 h. The supernatant bacterial solution was discarded, the bottom of the plates was gently cleaned twice with aseptic phosphate-buffered saline (PBS), and 150 μL of formaldehyde was added for fixation for 12 h. After removing the formaldehyde, 150 μL of crystal violet dye was added. After staining for 12 h, 150 μL of 75% ethanol was added for decolorization, and the ethanol was discarded. After air drying at room temperature, OD595 was detected using an enzyme labeler (Thermo Fisher Scientific, USA). Then, the final RES concentrations of 1/4 MIC, 1/2 MIC, and 1 MIC with 30 mmol/L N-Acetylcysteine (NAC; Beyotime, China) were added to *Staphylococcus aureus* and tested with the same method after culture for 24 h and 36 h, respectively. There were six repetition holes per concentration, and the experiment was independently repeated three times.

### 2.6 Laser confocal detection

The final RES concentrations of 1/4 MIC, 1/2 MIC, 1 MIC, and 30 mmol/L NAC were added to *Staphylococcus aureus*, and the culture was incubated at 37°C for 12 or 24 h. Then, the bacterial solution was removed and the well plate was gently washed twice with sterile PBSto wash off the non-adhesive bacteria. Next, 3 μL of SYTO-9 dye was mixed in the LIVE/DEAD^®^ BacLight Bacterial Viability kit (Thermo Fisher Scientific, United States) with PBS per mL and added to the cleaned well plate, followed by incubation in a dark environment at room temperature for 15–20 min. The excitation wavelength of the laser confocal microscope (Nikon, Japan) was 480 nm, the emission wavelength was 500 nm, and the same area of each hole was observed and photographed in a dark environment.

### 2.7 Detection of PIA production

A sample of 10 μL of the *Staphylococcus aureus* bacteria solution was added to Congo red plates containing 1/8 MIC, 1/4 MIC, 1/2 MIC, and 1 MIC RES and cultured at 37°C for 24 h to observe the results. The experiment was repeated three times.

### 2.8 Detection of eDNA release

A 200 μL sample of *Staphylococcus aureus* was added to 24-well plates, followed by the addition of RES at concentrations of 1/8 MIC, 1/4 MIC, and 1/2 MIC. The well-plates were incubated at 37°C for 24 h. Then, 200 μL of 0.5 mol/L ethylenediaminetetraacetic acid (EDTA) was added to each well and pre-cooled for 1 h. The supernatant was then discarded, and 500 μL of TEN buffer (Tris–HCl 0.121 g, Na_2_EDTA·2H_2_O 0.074 g, NaCl 0.5844 g, 150 mL of deionized water) was added to resuspend the biofilm at the bottom of the well-plate, with pH adjusted to 8.0 using NaOH. The final volume was kept brought to 200 mL and the buffer was stored at room temperature. The supernatant was transferred to a pre-cooled sterile, enzyme-free centrifuge tube and centrifuged at 15,000 r/min at a low temperature for 5 min. The resulting supernatant was transferred to a new centrifuge tube. Next, 300 μL of TE buffer (prepared by mixing 10 mL of 1 mol/L Tris–HCl, 2 mL of 0.5 mol/L EDTA, and deionized water volume up to 100 mL) was added, and an equal volume of phenol, chloroform, and isoamyl alcohol mixture (ratio: 50%: 48%: 2%) was then added. The solution was refrigerated at 15,000 r/min for 10 min. The obtained supernatant was mixed with an equal amount of chloroform: isoamyl alcohol (24:1) solution and extracted again. The upper aqueous phase was collected, and three times the volume of ice, anhydrous ethanol, and 1/10 volume of sodium acetate were added at −20°C overnight. The samples were thawed on ice, centrifuged at a low temperature of 15,000 r/min for 10 min to obtain precipitation, washed with 70% ice ethanol, dried at room temperature, and finally dissolved in 20 μL TE buffer. The eDNA content was detected using a NanoDrop 2000 ultra-micro spectrophotometer (Thermo Fisher Scientific, USA). The experiment was repeated three times.

### 2.9 Detection of ROS generation

The final RES concentrations of 1/8 MIC, 1/4 MIC, 1/2 MIC, 1 MIC, and 30 mmol/L NAC were added to *Staphylococcus aureus*, and the culture was incubated at 37°C for 24 h. According to the CM-H2DCFDA kit (Beyotime, China) instructions, the ROS production was measured using an enzyme labeler (Thermo Fisher Scientific, United States), with an excitation wavelength of 480 nm and an emission wavelength of 525 nm. There were six repetition holes per concentration, and the experiment was independently repeated three times.

### 2.10 qRT-PCR analysis

A total RNA extraction kit (Beyotime, China) and high-capacity cDNA reverse transcription kit (Beyotime, China) were used to obtain total RNA and cDNA according to the manufacturer’s instructions. The Quantitative Real-Time Reverse Transcription Polymerase Chain Reaction (qRT-PCR) was performed using SYBR Premix Ex Taq™ (Takara, Japan) with a PCR Instrument (Applied Biosystems, France). All primer information is shown in Table 1. The 2^−ΔΔCT^ method was used to calculate the normalized relative expression.

**Table 1:** Primer sequences used for qRT-PCR.

Gene	Primer sequence (5′ → 3′)	Product size (bp)
*icaA*	F: CAACACATGGCAAGCGGTTCATAC R: TCGACGTTGGCTACTGGGATACTG	109
*icaB*	F: TCCAAAACGAAGTGAGTGGGTT R: AAACCCAGTCGCCGGTATTTT	103
*icaC*	F: GGAGACTATTGGAACGTTACCAGC R: TGCGTGCAAATACCCAAGATAACA	89
*icaD*	F: TGGTCAAGCCCAGACAGAGG R: GACACAAGATATAGCGATAAGTGCTGT	89
*cidA*	F: GGAACCCGCAGATGACGAAA R: ACTCGCTGATTGTCTGGTCGT	135
*cidB*	F: ACGCAACGGTCGTATGTTTAG R: TCAGCATGACGCCAGTTAATAC	105
*SarA*	F: TTTGCTTCAGTGATTCGTTTATTTACTC R: GTAATGAGCATGATGAAAGAACTGTATT	134
*NADPH1*	F: GGAACCCGCAGATGACGAAA R: ACTCGCTGATTGTCTGGTCGT	135
*NADPH2*	F: GGAACCCGCAGATGACGAAA R: ACTCGCTGATTGTCTGGTCGT	146
*16S rRNA*	F: CAACCGTGGAGGGTCATT R: TCGCACATCAGCGTCAGT	110

### 2.11 Statistical analysis

A one-way analysis of variance followed by Tukey’s post-hoc test was used for groups’ comparisons using the Statistical Package for the Social Sciences (SPSS) 22.0 software (SPSS Incorporated, United States). The results are shown as mean ± standard deviation (mean ± SD). *p < 0.05 and **p-value of < 0.01 indicates significance compared to the control groups.

## 3 Results

### 3.1 Bacteriostatic effects of RES and three drugs against *Staphylococcus aureus*

**Figure 1:**
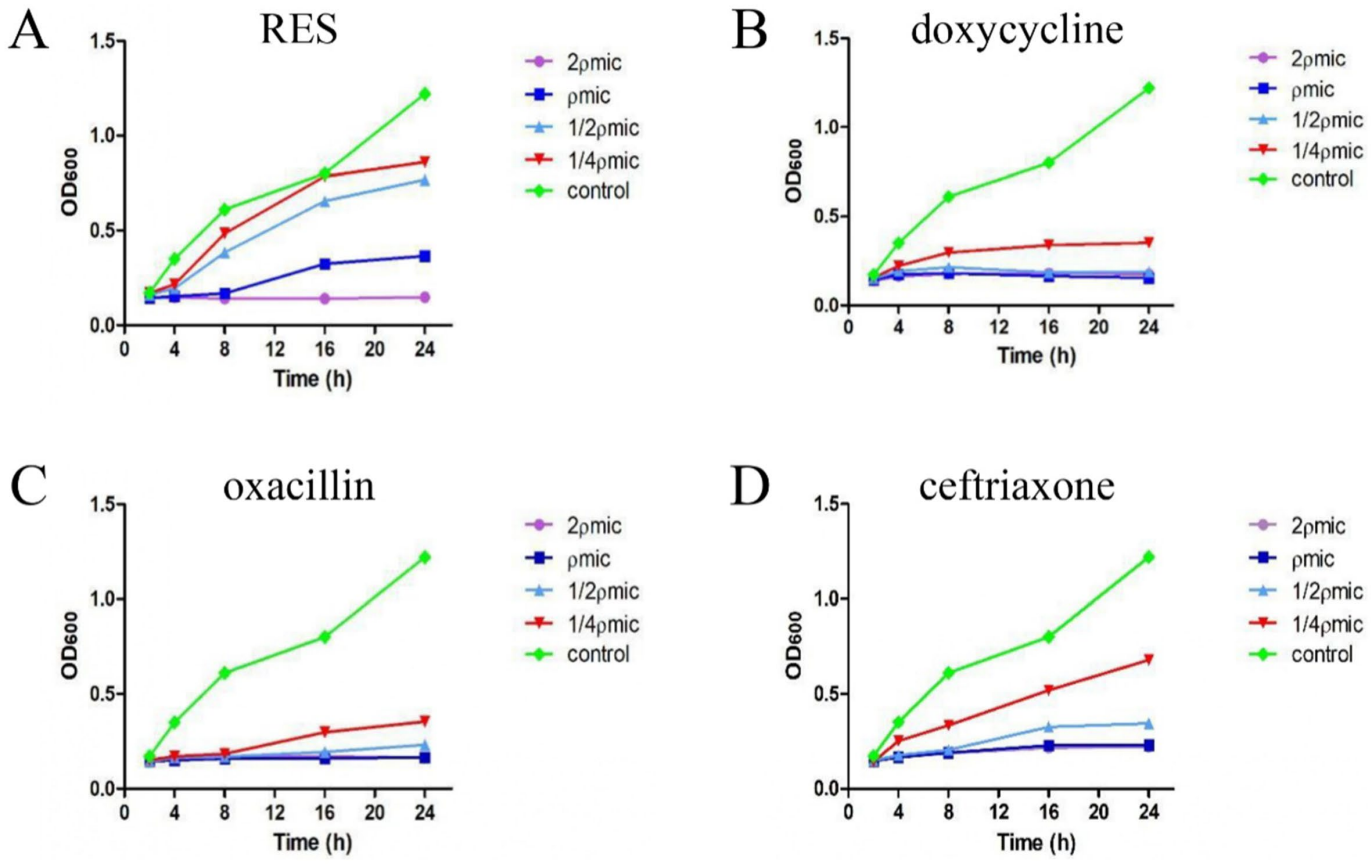
The inhibitory effect of RES and the three drugs on *Staphylococcus aureus*. **(A)** RES bactericidal curve (*n* = 3). **(B)** Doxycycline bactericidal curve (*n* = 3). **(C)** Oxacillin bactericidal curve (*n* = 3). **(D)** Ceftriaxone bactericidal curve (*n* = 3). Data are represented as means ± SD.

The MIC values of RES, doxycycline, oxacillin, and ceftriaxone against *Staphylococcus aureus* were 128 μg/mL, 16 μg/mL, 1.75 μg/mL, and 8 μg/mL, respectively. The inhibition curve showed that, when the RES concentration was 2ρMIC, the OD600 value exhibited a decreasing trend within 24 h, indicating that the number of bacteria showed a decreasing trend, and the trend gradually levelled off when the RES concentration was ρMIC. When the RES concentration was 1/2ρMIC and 1/4ρMIC, the OD600 value gradually increased after 8 h, indicating that the number of bacteria showed an increasing trend, and the number of bacteria gradually levelled off again after 24 h (Figure 1A). Within 24 h, doxycycline and oxacillin had significant inhibitory effects on *Staphylococcus aureus* at 1/4ρMIC concentration (Figures 1B,C). Ceftriaxone is less effective against *Staphylococcus aureus* than oxacillin and doxycycline but is still superior to RES (Figure 1D). These results indicate that the inhibitory effect of RES on *Staphylococcus aureus* is lower than that of the three drugs, but it still has a significant effect.

### 3.2 RES inhibited the formation of *Staphylococcus aureus* biofilms

**Figure 2:**
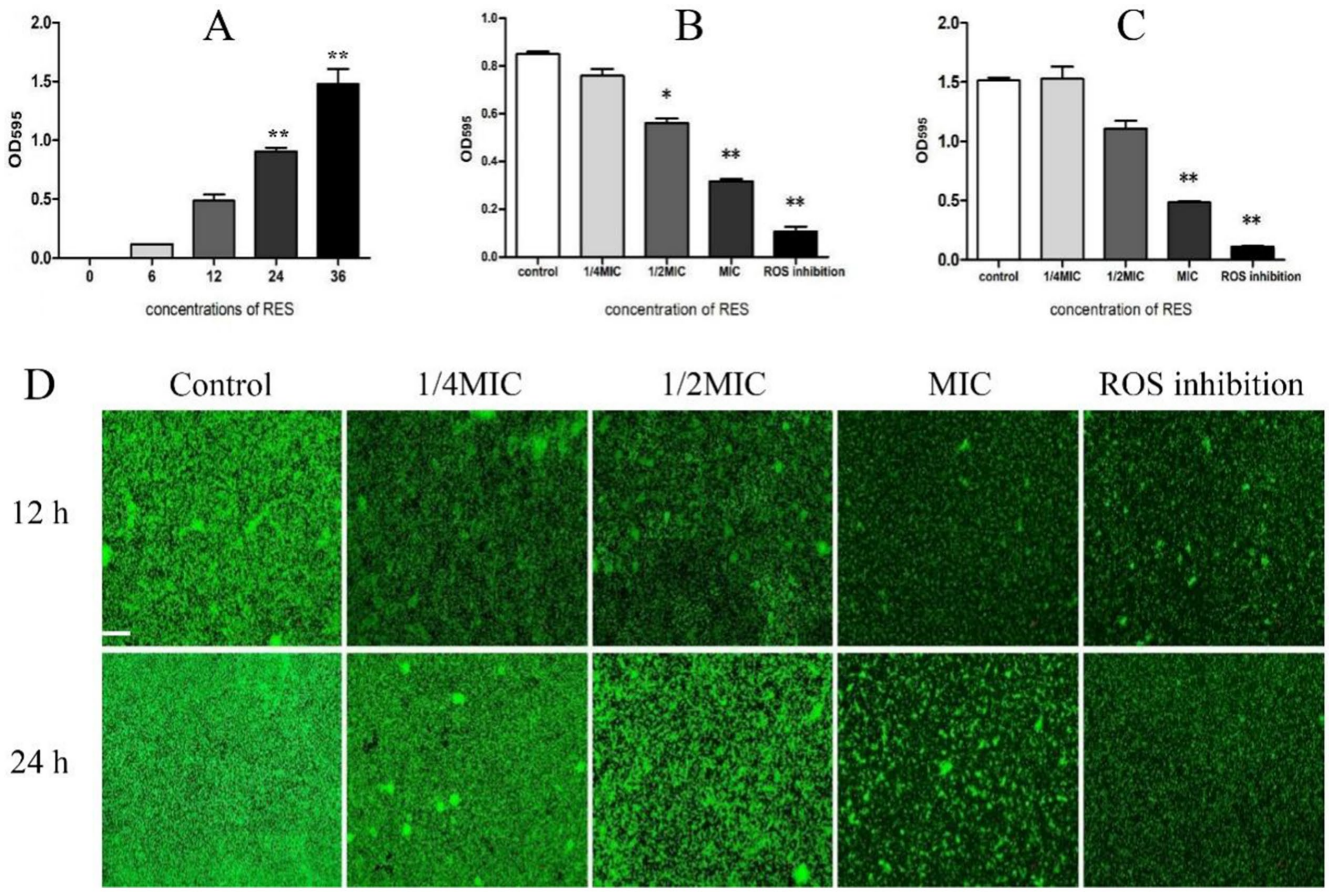
Effect of RES on biofilm formation of *Staphylococcus aureus*. **(A)** Detection of *Staphylococcus aureus* biofilm formation time (*n* = 3). **(B)** Effect of RES on the biofilm formation time of *Staphylococcus aureus* at 24 h (*n* = 3). **(C)** Effect of RES on the biofilm formation time of *Staphylococcus aureus* at 36 h (*n* = 3). **(D)** Representative image of laser confocal detection. Scale bar: 100 ×. *p < 0.05 and **p < 0.01 indicate significance compared to the control group.

The formation of *the Staphylococcus aureus* biofilm was stages. The results showed that biofilm formation was minimal between 0 and12 h but increased significantly after 24 h and 36 h (Figure 2A). This study then examined the effect of RES on *Staphylococcus aureus* biofilm formation. The results showed that, compared with the Control group, the biofilm formation ability of *Staphylococcus aureus* treated with MIC RES and ROS inhibitors was significantly inhibited. At 1/2 MIC concentration, biofilm formation was inhibited at 24 h but not at 36 h. There was no significant difference in biofilm formation between the 1/4 MIC RES treatment and Control groups (Figures 2B,C). Laser confocal detection results showed that, compared with the Control group, the thickness of *the Staphylococcus aureus* biofilm treated with RES was reduced. When RES was 1/2 MIC and 1 MIC, its effect was more pronounced, and a large number of pores and cavities appeared in *the Staphylococcus aureus* biofilm, especially when the RES concentration was 1 MIC, where the biofilm became fragmented. It was almost impossible for the biofilm to remain intact. In addition, the biofilm structure of *Staphylococcus aureus* strains treated with ROS inhibitors was significantly fragmented, and fluorescence intensity was reduced (Figure 2D). These results indicate that RES has a significantly inhibitory effect of *Staphylococcus aureus* biofilm formation.

### 3.3 RES inhibited the expression of factors related to biofilm formation

**Figure 3:**
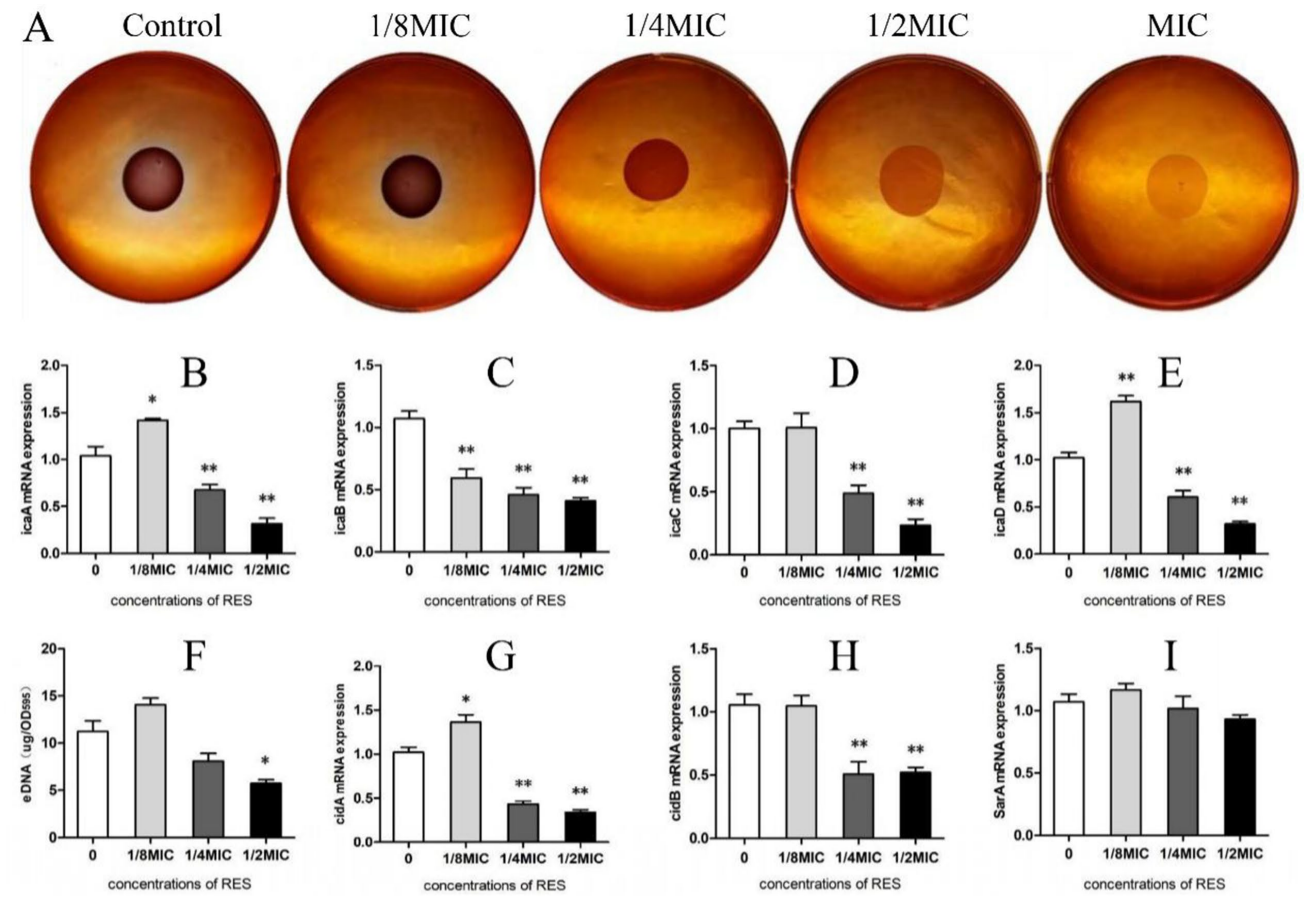
Effects of RES on factors related to *Staphylococcus aureus* biofilm formation. **(A)** PIA release detection. **(B)** *icaA* mRNA transcription level (*n* = 3). **(C)** *icaB* mRNA transcription level (*n* = 3). **(D)** *icaC* mRNA transcription level (*n* = 3). **(E)** *icaD* mRNA transcription level (*n* = 3). **(F)** Detection of eDNA production (*n* = 3). **(G)** *cidA* mRNA transcription level (*n* = 3). **(H)** *cidB* mRNA transcription level (*n* = 3). **(I)** *SarA* mRNA transcription level (*n* = 3). Data are represented as means ± SD. *p < 0.05 and **p < 0.01 indicate significance compared to the Control group.

When *Staphylococcus aureus* was inoculated with a Congo red plate for 24 h and the colony color was black, the PIA result was positive. If the colony color was light red, the PIA result was negative. The results showed that, when the RES concentration was 1/8 MIC, the color of the colony was black, which was darker than that of the Control group. With increasing RES concentration, the color of the colony gradually became lighter, and the amount of PIA synthesis decreased, indicating that RES inhibited the formation of PIA in *the Staphylococcus aureus* biofilm (Figure 3A). The detection results of the transcription levels of PIA-related genes showed that, compared with the Control group, when the RES concentration was 1/4 and 1/2MIC, the transcription levels of all genes measured decreased significantly. However, when the concentration of RES was 1/8 MIC, the transcription levels of *icaA* and *icaD* were higher than those of the Control group, whereas the transcription levels of *icaC* were not significantly different from those of the Control group. Only *icaB* transcription was inhibited at this concentration (Figures 3B–E). The results of eDNA production showed that a 1/2 MIC of RES significantly inhibited the release of eDNA from *Staphylococcus aureus*. There was no significant difference at a 1/4 MIC, and eDNA release at a 1/8 MIC was higher than that in the Control group (Figure 3F). The results of the detection of eDNA-related gene transcription levels were consistent with the results of eDNA production (Figures 3G,H). The SarA promotes adhesion and early biofilm formation by inhibiting nucleic acid cleavage and extracellular enzymes. The results of this study showed that the transcription level of the *SarA* gene in *Staphylococcus aureus* after RES treatment showed a downward trend, but the difference was not significant compared with that in the Control group (Figure 3I). These results indicate that RES inhibits the expression of most factors related to *Staphylococcus aureus* biofilm formation.

### 3.4 RES decreased ROS production in *Staphylococcus aureus*

**Figure 4:**
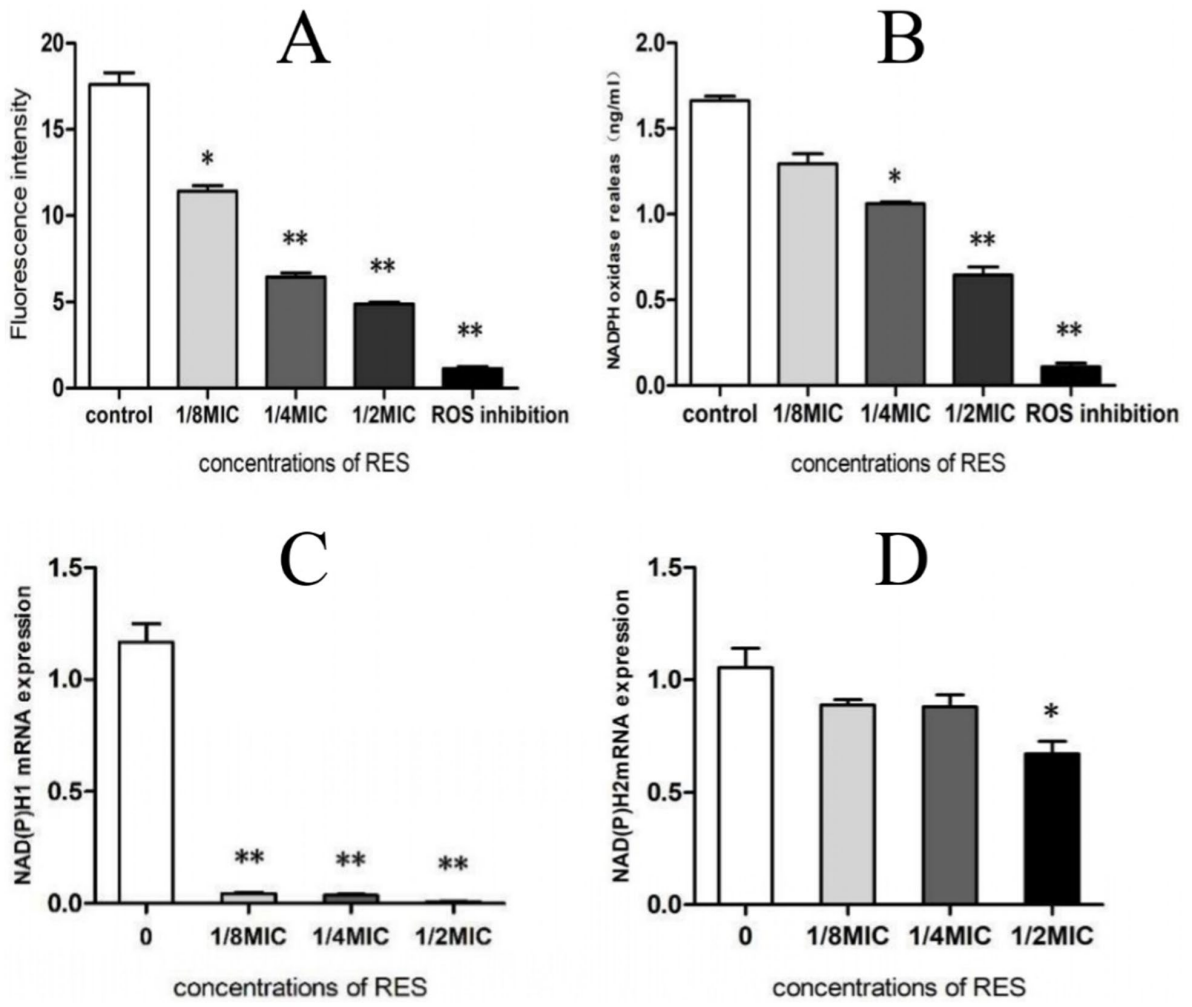
Effect of RES on ROS production by *Staphylococcus aureus*. **(A)** ROS generation (*n* = 3). **(B)** NADPH production (*n* = 3). **(C)** *NADPH1* mRNA transcription level (*n* = 3). **(D)** *NADPH2* mRNA transcription level (*n* = 3). Data are represented as means ± SD. *p < 0.05 and **p < 0.01 indicate significance compared to the control group.

The test results of ROS production showed that, compared with the Control group, RES treatment reduced the ROS production of *Staphylococcus aureus* in a dose-dependent manner; even if the concentration was 1/8 MIC, there was a significant difference (Figure 4A). The results of NADPH production showed that *Staphylococcus aureus* treated with RES at 1/4 MIC and 1/2 MIC exhibited a significant decrease in NADPH production compared with the Control group (Figure 4B). Compared with the Control group, the transcription of *NADPH1* was strongly inhibited after treatment of *Staphylococcus aureus* with all RES concentrations, whereas the expression level of *NADPH2* was significantly decreased only after treatment with 1/2 MIC RES (Figures 4C,D). These results suggest that RES can significantly inhibit ROS production by *Staphylococcus aureus*.

## 4 Discussion

*Staphylococcus aureus* is an important zoonotic pathogen with a complex immune escape mechanism (23) that easily produces a variety of antibiotic resistance, which poses great challenges to clinical treatment. RES, as a natural product with various biological activities, has been widely studied in recent years. In this study, the good inhibitory effect of RES against *Staphylococcus aureus* was also verified. However, a significant gap still exists between the antibacterial effect of RES and that of the three commercial drugs, which can be directly observed from the MIC values of each drug against *Staphylococcus aureus*. In addition, the antibacterial curve shows that RES had no inhibitory effect on *Staphylococcus aureus* at lower concentrations. Therefore, RES should be considered more as an adjuvant or adjunct to antibiotics.

In order to clarify the mechanism by which RES inhibits *Staphylococcus aureus*, we first examined the biofilm formation. The results of this study showed that the growth time of *the Staphylococcus aureus* biofilm was relatively long and that the amount of biofilm formed before 24 h was very low. The growth trend of the biofilm accelerated from 24 h to 36 h, and the biofilm reached maturity at 36 h. Next, the effect of RES on the biofilm formation of *Staphylococcus aureus* was studied. The results showed that the formation of the *Staphylococcus aureus* biofilm was significantly inhibited after treatment with RES at 1/2 MIC and 1 MIC for 24 h. However, under the 36-h culture conditions, only the 1 MIC of RES can significantly inhibit biofilm formation. It was hypothesized that the extended incubation time of 36 h led to medium evaporation, which may have increased the variability of the test results. Next, the experiment focused on two earlier time points at 12 h and 24 h to observe the effect of RES on the formation of *Staphylococcus aureus* biofilm by laser confocal microscopy. The results showed that both 1/2 MIC and 1 MIC of RES could cause a large number of pores and cavities in *a Staphylococcus aureus* biofilm and could not form a dense network structure, and the fluorescence intensity was low, indicating a significant impact on biofilm formation. However, the inhibitory effect at 12 h was still better than that at 24 h. Based on the above analysis, the effect of RES was short, which is consistent with the low bioavailability of RES reported by Cui et al. (24). If *Staphylococcus aureus* infection occurs, RES should be used immediately.

The process of bacterial biofilm formation is very complicated and involves the expression and regulation of many protein genes. PIA plays a fundamental role in mediating bacterial intercellular adhesion and is one of the important mechanisms in bacterial biofilm formation (25), which is regulated by the ica locus (26). A previous study showed that PIA promotes intercellular adhesion through polyvalent electrostatic interactions with polyanionic teichoic acid on the surface of *Staphylococcus aureus* cells (27). To investigate the effect of RES on the release of PIA from *Staphylococcus aureus*, we tested it by inoculation on a Congo red plate. The results showed that 1/4 MIC, 1/2 MIC, and 1 MIC of RES could reduce the release of PIA in a dose-dependent manner, indicating that RES inhibited the synthesis of PIA in the *Staphylococcus aureus* biofilm. Surprisingly, the color of *Staphylococcus aureus* colonies deepened after treatment with 1/8 MIC of RES, indicating that this concentration of RES may promote the synthesis of *Staphylococcus aureus* biofilm PIA. Then, qRT-PCR was used to determine the transcription level of the ica locus operon, and the results showed that a higher concentration of RES inhibited the transcription of all four genes, but when RES was 1/8 MIC, the transcription level of all four genes showed an overall increasing trend, which was consistent with the results of the Congo red plate experiment. We speculate that this finding may be correlated with the positive effects of natural polyphenols on biological organisms (28). However, RES effectively inhibited PIA release, which is consistent with the results reported by Qin et al., that RES interferes with the expression of genes associated with surface and secreted proteins and capsular polysaccharides (29). These findings suggest that resveratrol may be useful as an adjunct therapy for biofilm-associated *Staphylococcus aureus* infections.

eDNA is a crucial nucleic acid component of biofilms. Rice et al. found that the absence of the active gene *cidA* can lead to a reduction in bacterial cleavage and biofilm attachment, while reducing the amount of eDNA in these biofilms, indicating that eDNA plays a vital role in the early stages of biofilm formation (30). In this study, the amount of eDNA released by *Staphylococcus aureus* after RES treatment at 24 h was determined. Compared with the Control group, the amount of eDNA released by *Staphylococcus aureus* decreased after treatment with a higher concentration of 1/2 MIC RES, indicating that RES had a significant inhibitory effect on the eDNA release of *Staphylococcus aureus* at this concentration. The *cidA* and *cidB* gene transcription levels also declined. However, at the 1/8MIC RES concentration, the release of eDNA and the transcription of the two genes showed an increasing trend. This phenomenon was consistent with the detection results of PIA and its related genes at this concentration.

The SarA protein family, a class of DNA-binding proteins homologous to SarA, promotes adhesion and early biofilm formation by inhibiting nucleic acid cleavage and extracellular enzyme activity and is also a regulator of toxic gene expression in *Staphylococcus aureus*. Its homologs also play a similar role in other subspecies, including *Staphylococcus epidermidis*, *Staphylococcus haemolyticus*, and *Staphylococcus saprophyticus* (31). In this study, the effect of RES on the coregulator SarA of *Staphylococcus aureus* showed that the transcription level of the *SarA* gene showed a downward trend, but it was not significant compared with the Control group. It is speculated that this gene does not play a key role in the inhibition of biofilm formation by RES, but further research is still needed to confirm this speculation.

ROS is a general term for a class of molecules with oxidative activity produced by cells during energy metabolism under aerobic conditions. It not only plays an important role in the physiological processes of animals and plants but also plays a key role in the study of antibiotic sterilization and the generation of bacterial resistance (32–34). Due to the differences in the sources and types of ROS, they can have different effects on bacteria. For example, ROS produced by plants and animals due to bacterial infection can have a killing effect on pathogenic microorganisms, whereas ROS produced by bacteria during their own growth and reproduction can guarantee the survival of bacteria and promote the formation of biofilms (35). NADPH, as an oxidase, was positively correlated with ROS production (36). In this study, the ROS production was significantly reduced after the co-culture of *Staphylococcus aureus* and RES, and the expression level of NADPH was also decreased by enzyme-linked immunosorbent assay (ELISA), and both showed a gradient-dependent relationship. The transcription levels of NADPH-related genes were further detected, and the transcription levels of the two genes tested were downregulated. The formation of *Staphylococcus aureus* biofilms has a strong correlation with ROS and NADPH.

In this study, the antibiofilm effect of RES was confirmed using the standard strain of *Staphylococcus aureus* (ATCC 25923). However, it is important to note that significant differences may exist between the standard strain and clinical isolates, especially MRSA. Therefore, follow-up studies will be extended to include multiple clinical isolates and drug-resistant strains to assess the generalizability of these findings. In addition, the current *in vitro* results require further validation using animal models, such as mouse models of chronic wound infection or duct-associated biofilms, to evaluate the effects of local application of RES-based nanomaterials, such as liposome-encapsulated formulations, on biofilm clearance and host inflammatory response (37). In response to the low oral bioavailability of RES (38), novel drug delivery methods, such as aerosol inhalation (39) or sustained-release patches (40), should be explored to improve targeting. In terms of clinical translation, future research should prioritize evaluating the synergistic effects of RES in combination with antibiotics, particularly its potential role as an adjunctive therapy in treating MRSA infections.

## 5 Conclusion

In summary, we found that RES inhibited the formation of *Staphylococcus aureus* biofilms by reducing PIA, eDNA release, and ROS production. The addition of RES may be an effective treatment for *Staphylococcus aureus* infection.

## Data Availability

The original contributions presented in the study are included in the article/supplementary material, further inquiries can be directed to the corresponding author.
